# STRESS granule-associated RNA-binding protein CAPRIN1 drives cancer progression and regulates treatment response in nasopharyngeal carcinoma

**DOI:** 10.1007/s12032-022-01910-w

**Published:** 2022-12-14

**Authors:** Te Yang, Long Huang, Haide Qin, Shijuan Mai

**Affiliations:** 1 Guangdong Provincial Key Laboratory of Malignant Tumor Epigenetics and Gene Regulation, Sun Yat-Sen Memorial Hospital, Sun Yat-Sen University, Guangzhou, 510120 China; 2 State Key Laboratory of Oncology in South China, Guangdong Key Laboratory of Nasopharyngeal Carcinoma Diagnosis and Therapy, Sun Yat-Sen University Cancer Center, Guangzhou, 510060 China

**Keywords:** Nasopharyngeal carcinoma (NPC), RNA-binding protein, Stress granule, *CAPRIN1*, mTOR

## Abstract

Nasopharyngeal carcinoma (NPC) is a common malignancy of the head and neck that is mainly diagnosed in southern China and Southeast Asia, with a strong etiological link to Epstein‒Barr virus infection. Those with advanced-stage disease have a significantly worse prognosis. There is an urgent need to identify novel therapeutic targets for the recurrent or metastatic nasopharyngeal carcinoma. With a particular focus on Cell Cycle Associated Protein 1 (CAPRIN1), one of the important RNA-binding proteints associated with stress granule formation, we used RT‒qPCR and immunohistochemistry to validate CAPRIN1 expression in NPC tissues and cell lines. Further, CAPRIN1 expression was knocked down using siRNA, and the effect on cell proliferation and migration was systematically assessed by in vitro assays. As a result, we demonstrated that CAPRIN1 was elevated in NPC compared to adjacent normal tissues. Knockdown of CAPRIN1 in NPC cells inhibited proliferation and migration, involving the regulation of cell cycle protein CCND2 and EMT signaling, respectively. Notably, we found that CAPRIN1 knockdown promoted cell apoptosis by regulation of the expression of apoptosis-related proteins cleaved-PARP and cleaved-Caspase3. Knockdown of CAPRIN1 increased NPC cell sensitivity to rapamycin, and increased NPC cell sensitivity to cisplatin and to X-rays. In conclusion, CAPRIN1 might drive NPC proliferation, regulate cell cycle and apoptosis, and affect tumor cell response to anti-cancer agents and X-ray irradiation. CAPRIN1 might serve as a potential target for NPC.

**Supplementary Information:**

The online version contains supplementary material available at 10.1007/s12032-022-01910-w.

## Introduction

Nasopharyngeal carcinoma (NPC) is a common type of head and neck cancer that is highly prevalent in the southern and southeastern regions of China [1]. There were 129,000 new cases of NPC in 2018, primarily in East and Southeast Asia [2]. According to the WHO, the nonkeratinizing-type of NPC accounts for more than 95% of cases in southern China [3] and is mainly associated with EBV infection [4, 5]. Plasma viral load could predict outcomes and monitor disease progression [6]. The pathogenesis of NPC and its prognostic factors are less well understood.

Clinically, approximately 10~20% of patients with NPC experience relapse and treatment failure after radiotherapy. Although targeted therapies and PD-1 monoclonal antibody-based immunotherapy have shown efficacy for recurrent/metastatic nasopharyngeal cancer [7], the development of drug resistance and the limited indicated subpopulation remain challenges for anti-cancer agents [8, 9]. Previous studies have shown that combinations of chemotherapeutic agents can improve the outcome of patients with relapsed or metastatic NPC [10, 11], but little is known about the rationale for combination regimens, and the mechanisms underlying disease progression are not yet clear. Therefore, it is important to search for more effective therapeutic targets and discover novel biomarkers for the screening of appropriate patients in the context of NPC.

At present, radiotherapy is the main treatment for NPC. The advent of new radiotherapy technologies such as intensity-modulated radiotherapy (IMRT) has achieved good responses for nasopharyngeal carcinoma [1]. Moreover, previous studies have shown that combination therapies with radiotherapy and chemotherapeutic agents can improve the treatment response for local-regionally advanced nasopharyngeal carcinoma [12]. Although the methods of radiotherapy and chemotherapy for NPC have improved in recent years, there remain challenges in improving the radiotherapy effect on NPC.

Previous studies have shown that RNA-binding proteins (RBPs) have the functions of regulating treatment response and tumor cell proliferation [13], and could be potential as targets for anti-cancer therapies. Among the human RBPs, there is a unique subset of RBPs such as G3BP1-associated proteins can recruit RNAs to promote the assembly of stress granules (SGs) to regulate translation initiation when cells exposed to the increase of reactive oxygen species (ROS) and oxidative stress in cells [14]. The formation of SGs regulates cellular stress response in pathological conditions [13], including tumor progression and treatment response [15]. It has also been reported that stress particles play roles in hepatocellular carcinoma (HCC) [16] and colorectal cancer [17] et al. However, the role and mechanism of stress granule formation within the context of oxidative stress and the effects on radiotherapy and chemotherapy for NPC are still unclear.

In this study, we reported one of important RNA-binding proteins related to SGs, CAPRIN1 (cell cycle associated protein 1), as a potential target for NPC. Using the in vitro experiments, we systematically characterized the functions and mechanisms of CAPRIN1 in NPC. Our results showed that CAPRIN1 might play important roles in promoting NPC proliferation, regulating apoptosis, and affecting the response to radiotherapy and chemotherapy. Molecular characterization of CAPRIN1 in NPC will be helpful for clarifying the roles of a variety of RNA-binding proteins in cancer.

## Methods

### Cell lines

The cell lines used in this study included the NPC cell lines HK-1, CNE1, CNE2, 5-8F, 6-10B and SUNE1. All cell lines were stored in the laboratory and cultured in RPMI 1640 complete medium (RPMI 1640 supplemented with 10% fetal bovine serum) (Gibco) at 37 °C in an atmosphere containing 5% CO_2_; immortalized nasopharyngeal epithelial NP69 cells were cultured in keratinocyte-serum free medium (KFSM) containing EGF and bovine pituitary extract (Gibco) at 37 °C and 5% CO_2_.

### siRNA transfection

siRNA was synthesized by RiboBio. For siRNA transfection, the cells were cultured in six-well plates. siRNA and LIPO3000 Liposome Transfection Reagent (Invitrogen, American) were separately added to Opti-MEM and then incubated for 5 min; these two solutions were mixed and incubated at room temperature for 10 min. The transfected cells were incubated at 37 °C and 5% CO_2_ for more than 24 h.

### Protein extraction

For protein extraction, we used protease and phosphatase inhibitors from Thermo Fisher Scientific. PMSF and RIPA lysis buffer were obtained from Beyotime Company. The lysis buffer contained 1 mL RIPA lysis buffer, 1 μL protease inhibitor, 5 μL PMSF, and 10 μL phosphatase inhibitor. For cells in the logarithmic growth stage, the culture medium was discarded, the cells were washed with normal saline, lysis buffer was added, and the cells were considered fully lysed after incubation on ice for 10 min. The liquid was collected into a clean EP tube at 4 °C and centrifuged at 14,000×*g* for 20 min. The supernatant was transferred to another clean EP tube, and protein was stored at − 80 °C.

### Protein quantification and denaturation

The BCA protein quantification kit from Beyotime Company was used. Briefly, 200 μL BCA working solution was added to each well of a 96-well plate; then, deionized water and BSA standard protein were added to the prepared BCA working solution according to the instructions to generate the standard curve; 19 μL deionized water and 1 μL protein were added to each well. After shaking the plate to mix the solutions, the plate was incubated at 37 °C for 30 min. The absorbance value at 562 nm was detected by a microplate reader. The standard curves were derived using the standard protein, and then, the protein concentration in samples was calculated according to the standard curve. After the sample protein was added to the lysis buffer and the loading buffer, the mixture was immediately vortexed. Then, the tube was heated in a metal bath at 100 °C for 10 min, and the sample was stored short-term at − 20 °C or long-term at − 80 °C.

### Clinical samples and Immunohistochemistry

Fresh tissues were obtained during surgical resection at the Sun Yat-sen University Cancer Center. Immunohistochemical staining was performed according to routine pathology laboratory operation. Briefly, paraffin-embedded tissue sections were heated at 60 °C overnight, immersed in xylene for dewaxing and then hydrated in a concentration gradient of ethanol and distilled water. Antigen repair was carried out in a pressure cooker for 2.5 min, and the sections were incubated with primary antibody overnight at 4 °C. After blocking with 3% hydrogen peroxide for 10 min, the sections were incubated with secondary antibody for 30 min at 37 °C. DAB was added for approximately 1 min, and then, the sections were quickly placed into water. The nuclei were stained with hematoxylin staining solution for 1–5 min, and the reaction was terminated in water. The sections were rinsed with 1% hydrochloric acid alcohol for 3 s and then with running water for more than 30 min. After the sections were dried, they were sealed with environmental protection resin sealing agent.

### Western blotting

For western blotting, SDS‒PAGE gels, electrophoresis buffer and transfer buffer were prepared. The proteins were separated by gel electrophoresis at 80 V in the concentrating gel, and then the voltage was adjusted to 120 V in the separating gel. PVDF membranes were soaked in methanol for 5–10 min, and then, protein transfer was performed according to the Bio–Rad semidry transfer instructions. The membranes were blocked with protein-free rapid blocking solution for 15 min. The membranes were incubated with primary antibody overnight at 4 °C on a shaker. Next, the membranes were incubated in secondary antibody for 1 h on a shaker at room temperature. Finally, ECL solution was added to the membrane, which was then exposed on a chemiluminescence imager.

### Cell proliferation assays

For the CCK-8 assay, a 96-well plate with 200 μL/well complete medium and 500–2000 cells per well was used. After the plates were incubated at 37 °C with 5% CO_2_ for 24 h, 100 μl CCK-8 working solution was added to each well, and 100 μL complete medium was added to a well as a blank control. The cells were incubated at 37 °C with 5% CO_2_ for 2 h, and then, the absorbance at 450 nm was measured using a microplate reader.

For the colony formation assay, 600–1000 cells/well were seeded into a six-well plate at 48 h after transfection. A total of 10–12 days later, the cells were fixed with methanol for 30 min and then stained with crystal violet (Beyotime, China).

### Transwell assay

For the Transwell assay, 8.0 µm polycarbonate membrane Transwell chambers (Coning) were placed into a 24-well plate with complete medium. A total of 30,000–100,000 cells were seeded into the chambers and incubated at 37 °C and 5% CO_2_. After 14–24 h of incubation, cells on the upper side of the membrane were removed. Cells on the lower side of the membrane were fixed with methanol for 10 min. The plate was washed once and stained with crystal violet for more than 30 min.

### Cell cycle assay

For the cell cycle assay, a total of 2 × 10^5^–5 × 10^5^ cells/well were seeded into six-well plates and cultured overnight. The cells were starved for 24 h, and then, siRNA knockdown experiments were conducted in complete medium. After 24–48 h of treatment, the cells were collected, and 1 mL DNA stabilizing solution and 10 μL permeabilization solution were added. The cells were incubated at room temperature in the dark for 30 min and then analyzed by flow cytometry based on the fluorescence at 488 nm.

### Apoptosis assay

For cell apoptosis analysis, cells in the logarithmic growth phase were collected by digestion with trypsin without EDTA and centrifuged. Then, 100 μL 1 × Binding buffer was used to suspend the cell pellet, and 5 μL Annexin V-Alexa Fluor 647 and 10 μL PI were added. The cells were incubated at room temperature in the dark for 15 min. Then, 400 μL 1 × binding buffer was added. The prepared samples were analyzed by flow cytometry.

### Drug sensitivity assay

For the drug sensitivity assay, 5000 cells were seeded per well in a 96-well plate and incubated overnight. We set up eight concentration gradients and prepared medium containing different concentrations of the drug in complete medium. The cell culture medium in the 96-well plate was replaced with drug-containing medium. After 24–48 h of drug treatment, cytotoxicity was determined using the CCK-8 method. Cell viability at different concentrations was estimated, and the half-maximal inhibitory concentration (IC50) of the drug was calculated.

### Radiotherapy sensitivity assay

Cells were collected after transfection with siRNA for 24 h. The cells were seeded in 6-well plates at 500, 1000, 2000, 5000, 10,000, and 20,000 cells/well and then irradiated at 0 Gy, 2 Gy, 4 Gy, 6 Gy, 8 Gy, and 10 Gy, respectively. The cells were cultured for approximately 14 days in a humidified incubator with 5% CO_2_ at 37 °C. After crystal violet staining, the cells were counted, and the cell survival fraction was calculated. Survival fraction = colony formation rate of treated cells/colony formation rate of untreated cells. Colony formation rate = number of colonies formed/number of cells plated.

### Lentiviral infection and construction of stable transgenic cell lines

The cells were seeded into 6-well plates with 2 mL complete medium. For the experimental group, 2 μL polybrene and 30–50 μL virus solution were added to each well. After the plates were incubated at 37 °C with 5% CO_2_ for 48 h, the virus-containing medium was discarded, and the experimental and control groups were incubated with complete medium containing 2 μg/ml puromycin to start the drug screen. In the control group, the cells were basically dead, confirming the successful construction of cells in the experimental group.

## Results

### CAPRIN1 expression is correlated with cellular stress and upregulated nasopharyngeal carcinoma

**Fig. 1:**
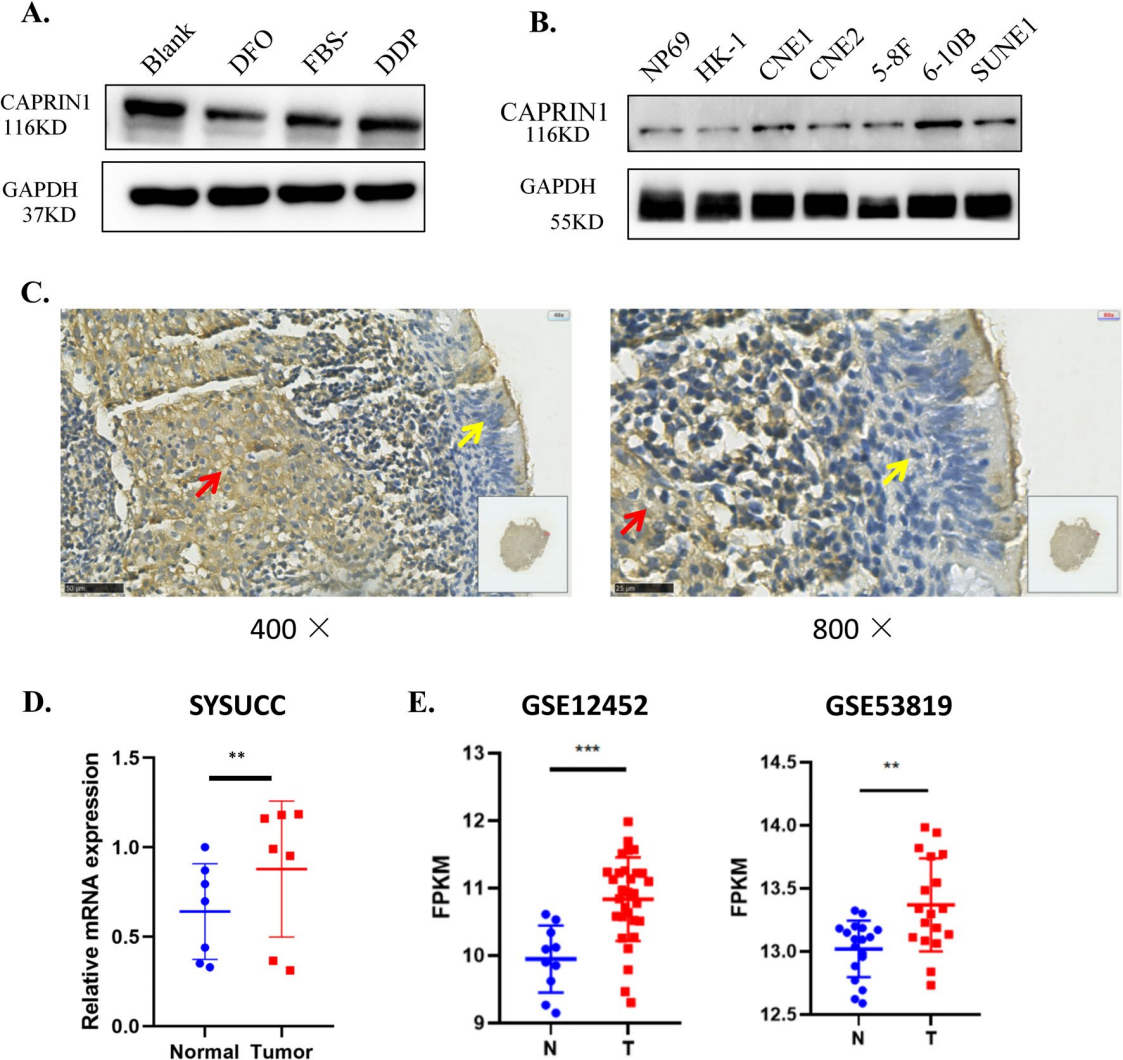
CAPRIN1 is associated with cellular stress and upregulated in NPC cells and tissues. **A** CAPRIN1 protein expression changed upon exposure to DFO, starvation, and cisplatin in 6-10B cells. **B** Protein expression of CAPRIN1 in NPC cells. **C** Representative images for the expression levels of CAPRIN1 in NPC tissues. Note, the red arrows indicated tumor cells; the yellow arrows indiated normal cells. **D** mRNA expression of CAPRIN1 in normal tissues versus NPC tissues. **E** CAPRIN1 expression in GEO datasets GSE12452 and GSE53819. **p < 0.01, ***p < 0.001

Previous studies have indicated that CAPRIN1 is part of stress granules, which are non-membrane cytoplasmic structures composed of mRNA and proteins that form when cells are exposed to various stress stimuli. To investigate whether CAPRIN1 responds to stress conditions in NPC cells, we examined CAPRIN1 protein levels by western blotting in 6-10B cells exposed to oxygen stress (100 μM DFO), starvation (no serum), and cisplatin stimulation (10 μM). We found that CAPRIN1 protein levels decreased after DFO treatment, starvation, and cisplatin stimulation (Fig. 1A). The results suggest that CAPRIN1 is an important response gene in stress conditions in NPC cells and might affect the response to treatment of NPC in chemotherapy and/or radiation therapy.

To characterize the functions of CAPRIN1 in NPC, we investigated the expression of CAPRIN1 in nasopharyngeal carcinoma cell lines and tissues. We detected CAPRIN1 mRNA expression by RT-qPCR and CAPRIN1 protein expression by Western blotting in NPC cell lines and found that CAPRIN1 was highly expressed in most NPC cell lines (Fig. 1B; Supplementary Fig. S1).

IHC assay also confirmed that the protein expression levels were elevated in tumor cells as compared to the normal epithelial cells in NPC tissues (Fig. 1C). By using RT-qPCR, we validated CAPRIN1 gene expression in seven NPC tissues and compared it to that in adjacent noncancerous tissues. We confirmed that CAPRIN1 expression was significantly elevated in NPC tissues compared to normal tissues (Fig. 1D).

Furthermore, data analysis of NPC microarray gene expression data showed that CAPRIN1 was significantly highly expressed in tumor tissue in two NPC gene microarray datasets (GSE12452 and GSE53819) (Fig. 1E). Moreover, the analysis of TCGA-HNSC RNA-Seq data showed that CAPRIN1 was consistently upregulated in head and neck tumors. (Supplementary Fig. S2).

### Knockdown of CAPRIN1 inhibits the proliferation of NPC cells

**Fig. 2:**
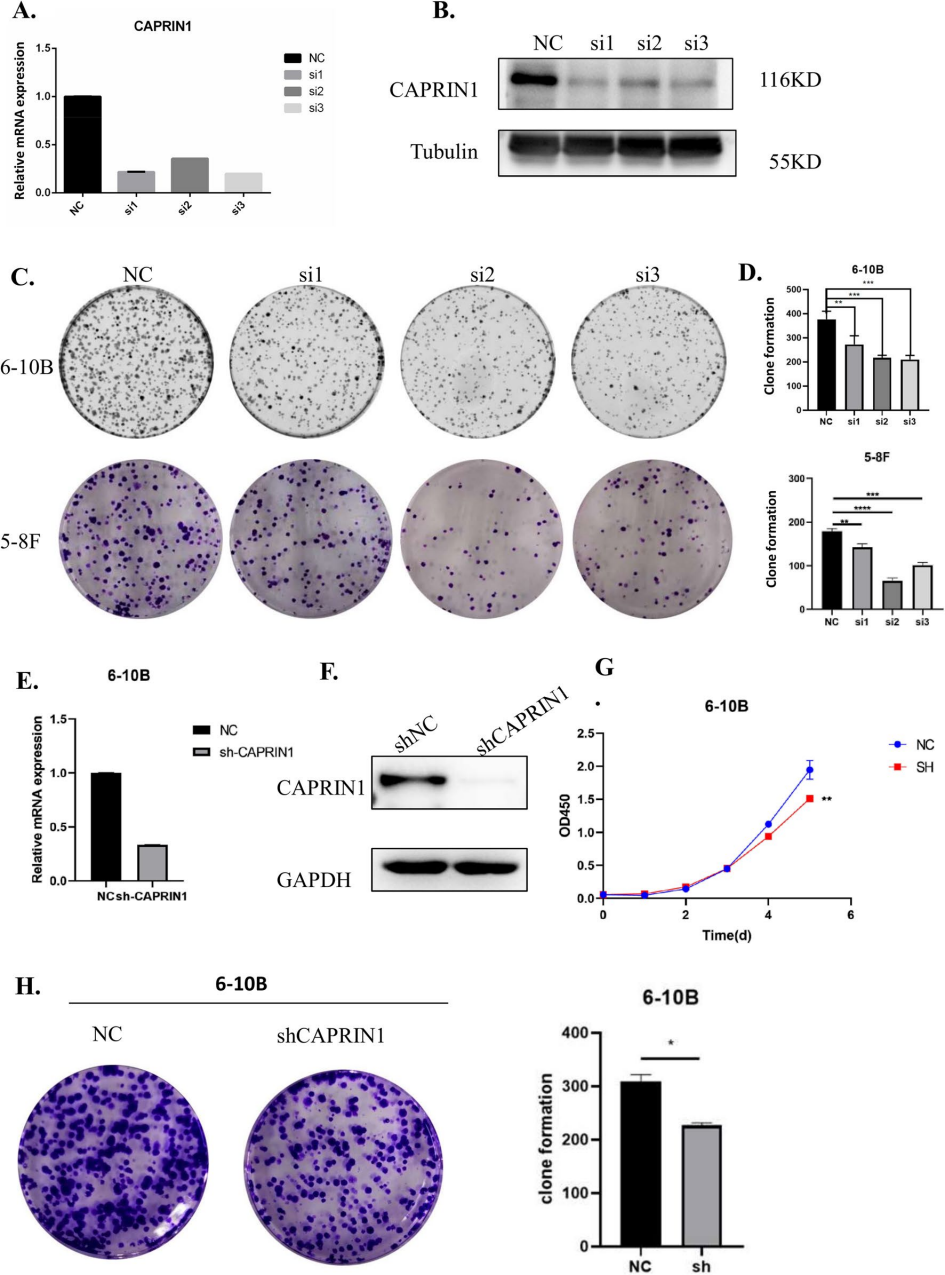
Knockdown of CAPRIN1 in NPC cells inhibits cell proliferation. **A** mRNA expression of CAPRIN1 after knockdown in 6-10B cells. **B** Protein expression of CAPRIN1 after knockdown in 6-10B cells. **C**, **D** Cell proliferation was detected by colony formation assays after CAPRIN1 knockdown in 6-10B and 5-8F cells. Note, *p < 0.05, **p < 0.01, ***p < 0.001. **E** mRNA expression of CAPRIN1 in 6-10B/shCAPRIN1 cells. **F** Protein expression of CAPRIN1 in 6-10B/shCAPRIN1 cells. **G** Cell proliferation detected by CCK-8 assay and colony formation assay (**H**) in 6-10B/shCAPRIN1 cells

To investigate the biological functions of CAPRIN1 related to cell proliferation in NPC, we knocked down CAPRIN1 in NPC cells by RNA interference (Fig. 2A, B). Then, we determined the proliferative capacity of NPC cells after CAPRIN1 knockdown using a CCK-8 assay and colony formation assay. Here, we selected two cell lines with good logarithmic growth activity. The results showed that the proliferative capacity was significantly reduced after CAPRIN1 knockdown in 6-10B and 5-8F cells, indicating that CAPRIN1 may be associated with NPC cell proliferation (Fig. 2C, D).

Furthermore, we established stable cell lines with CAPRIN1 knockdown using the NPC cell lines 6-10B. We performed RT‒qPCR and western blotting analyses of the stable cell lines and found that the mRNA and protein levels of CAPRIN1 were significantly reduced in 6-10B/shCAPRIN1 cells compared to those in parental 6-10B cells (Fig. 2E, F). CCK-8 and colony formation assays showed that 6-10B/shCAPRIN1 cells had a significantly lower proliferative capacity than 6-10B/shNC control cells, consistent with the results of the previous RNA interference assay (Fig. 2G, H).

### Knockdown of CAPRIN1 inhibits the cell cycle

**Fig. 3:**
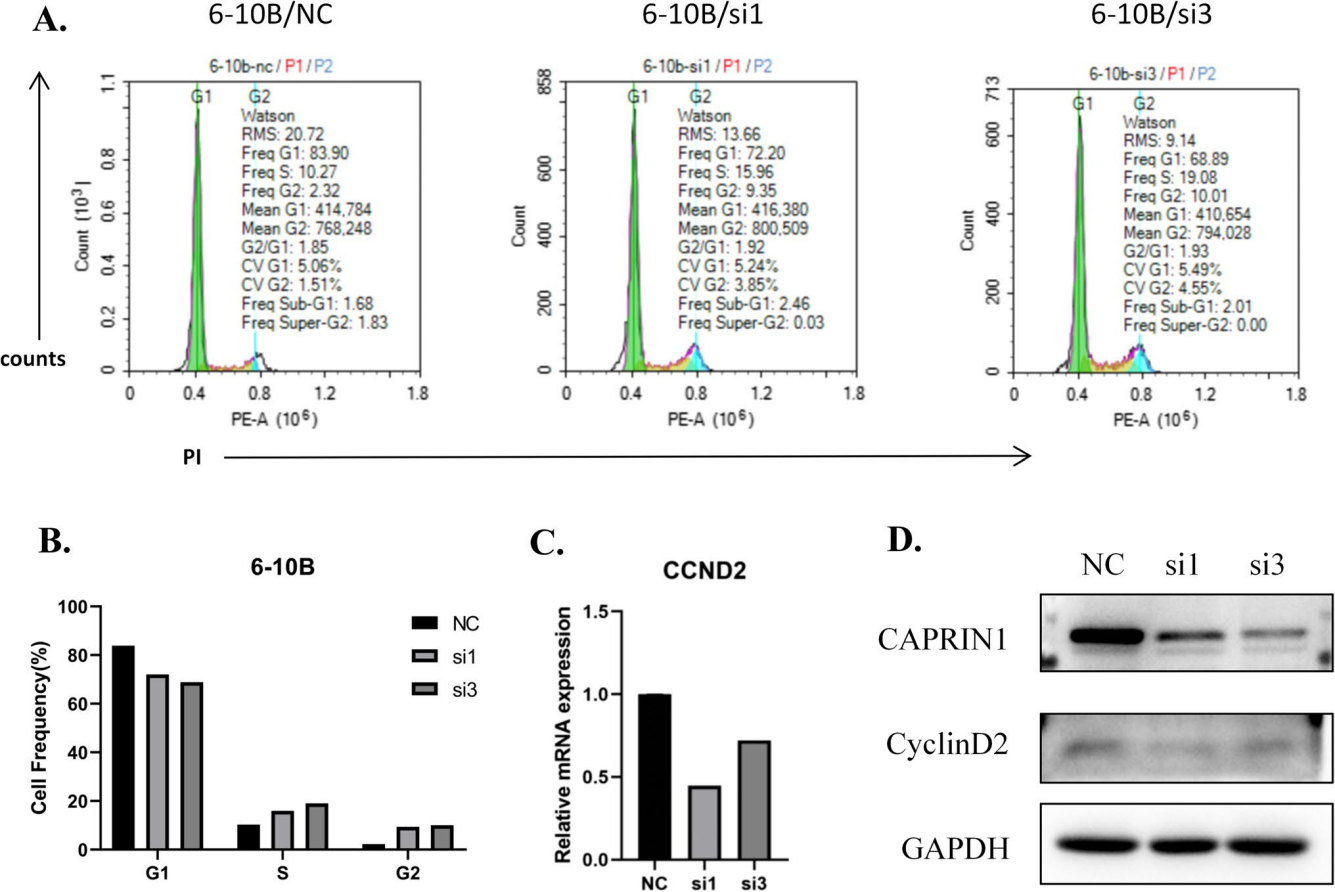
Knockdown of CAPRIN1 inhibits the cell cycle. **A** Flow cytometry analysis of 6-10B parental cells and CAPRIN1-knockdown cells. **B** Barplot for the Cell cycle changes after CAPRIN1 knockdown in 6-10B cells. **C** mRNA and **D** protein expression of Cyclin D2 after CAPRIN1 knockdown in 6-10B cells

To elucidate the mechanisms by which CAPRIN1 influences cell proliferation, we performed flow cytometry to examine cell cycle progression and apoptosis in NPC cells after CAPRIN1 knockdown. The results showed that the population of 6-10B cells in G0/G1 decreased and that in S phase increased after knockdown of CAPRIN1, suggesting that CAPRIN1 knockdown in NPC might block cells from exiting S phase (Fig. 3A, B).

In addition, CAPRIN1 has been reported to bind to mRNA, and Cyclin D2 (CCND2) is one of its targets [18]. Therefore, we examined the changes in CCND2 mRNA levels by RT‒qPCR after knocking down CAPRIN1 in 6-10B cells. The results showed that the mRNA and protein levels of CCND2 decreased after CAPRIN1 knockdown in 6-10B cells (Fig. 3C, D). Since CCND2 is an important cyclin protein in cancer cells, CAPRIN1 may promote cell proliferation by regulating the cell cycle through binding to CCND2 to induce its expression in NPC.

### Knockdown of CAPRIN1 promotes apoptosis and increases cell sensitivity to cisplatin and rapamycin

**Fig. 4:**
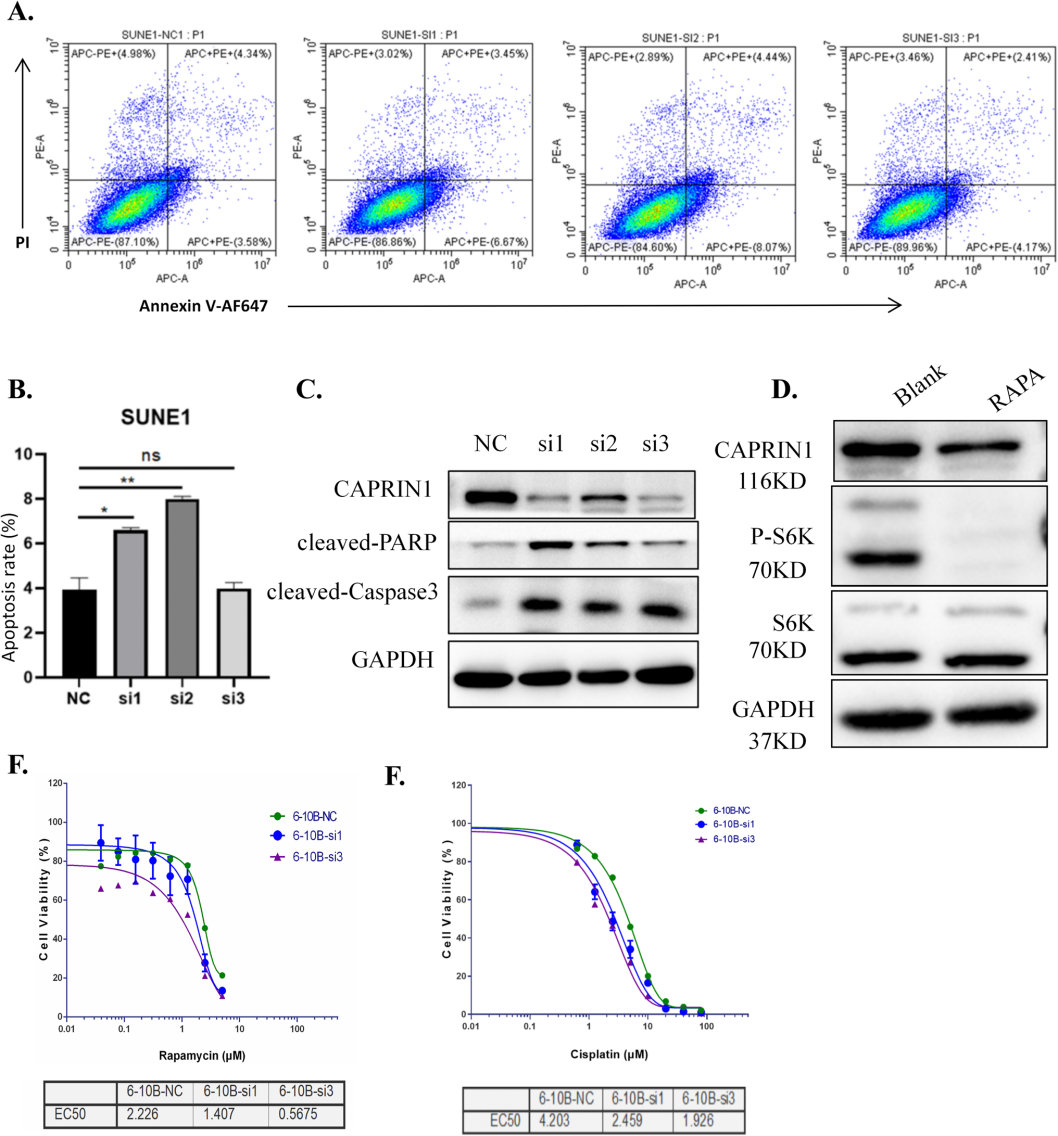
Knockdown of CAPRIN1 promotes cell apoptosis, and enhances sensitivity to cisplatin and rapamycin. **A** Apoptosis analysis of SUNE1 cells by flow cytometry after CAPRIN1 knockdown. **B** Statistical analysis of early-apoptosis among SUNE1 cells with CAPRIN1 knockdown compared with the parental cells. **C** Knockdown of CAPRIN1 induced the expression of apoptosis-related proteins cleaved-PARP and cleaved-Caspase3. **D** CAPRIN1 protein levels decreased after the addition of rapamycin to 6-10B cells. **E**, **F** The IC50 values of cisplatin and rapamycin in 6-10B cells decreased after CAPRIN1 knockdown

Previous studies suggest that S phase arrest might induce cell apoptosis. Therefore, we examined the effect of CAPRIN1 on apoptosis by flow cytometry. Here, we chose SUNE1 with high survival rate. In SUNE1 cells, early-apoptosis was increased by CAPRIN1 knockdown, as shown by the Annexin V-PI double-staining assay (Fig. 4A, B). Western blotting analysis of apoptosis-related proteins showed that CAPRIN1 knockdown increased activated PARP and Caspase 3 levels, indicating that CAPRIN1 inhibits apoptosis in NPC cells (Fig. 4C).

Based on the findings in correlation with cell apoptosis and our experiments in NPC cell lines that have showed that CAPRIN1 protein was down-regulated in the treatment of cisplatin (Fig. 1A), we speculated that CAPRIN1 might have effects on the drug response to mTOR inhibitors, which a promising anti-cancer agent targeting mTOR signaling that has shown efficacy in NPC in one of our previous studies [19]. Here, we used 6-10B cells in order to synchronize with the previous experiment. We examined the responses of CAPRIN1 upon exposure to rapamycin, and observed a decrease in CAPRIN1 protein levels in 6-10B cells treated with 500 nM rapamycin, suggesting that inhibition of the mTOR pathway also reduces CAPRIN1 protein levels (Fig. 4D). Our data suggest that CAPRIN1 is an important response gene in the drug treatment of NPC cells with cisplatin or rapamycin.

Furthermore, since both cisplatin and rapamycin can alter CAPRIN1 protein levels in NPC cells, we investigated the changes in the sensitivity of NPC cells to both cisplatin and rapamycin after CAPRIN1 knockdown. The 6-10B cell survival curves measured by CCK-8 assays after CAPRIN1 knockdown and drug stimulation are shown in (Fig. 4E, F). The IC50 values for both cisplatin and rapamycin in 6-10B cells decreased after CAPRIN1 knockdown. Taken together, our data suggest that CAPRIN1 knockdown might confer sensitivity to cisplatin and rapamycin in NPC cells, which may be useful for the clinical application of CAPRIN1 as a target in drug combinations.

### Knockdown of CAPIRN1 increases cell sensitivity to X-rays

**Fig. 5:**
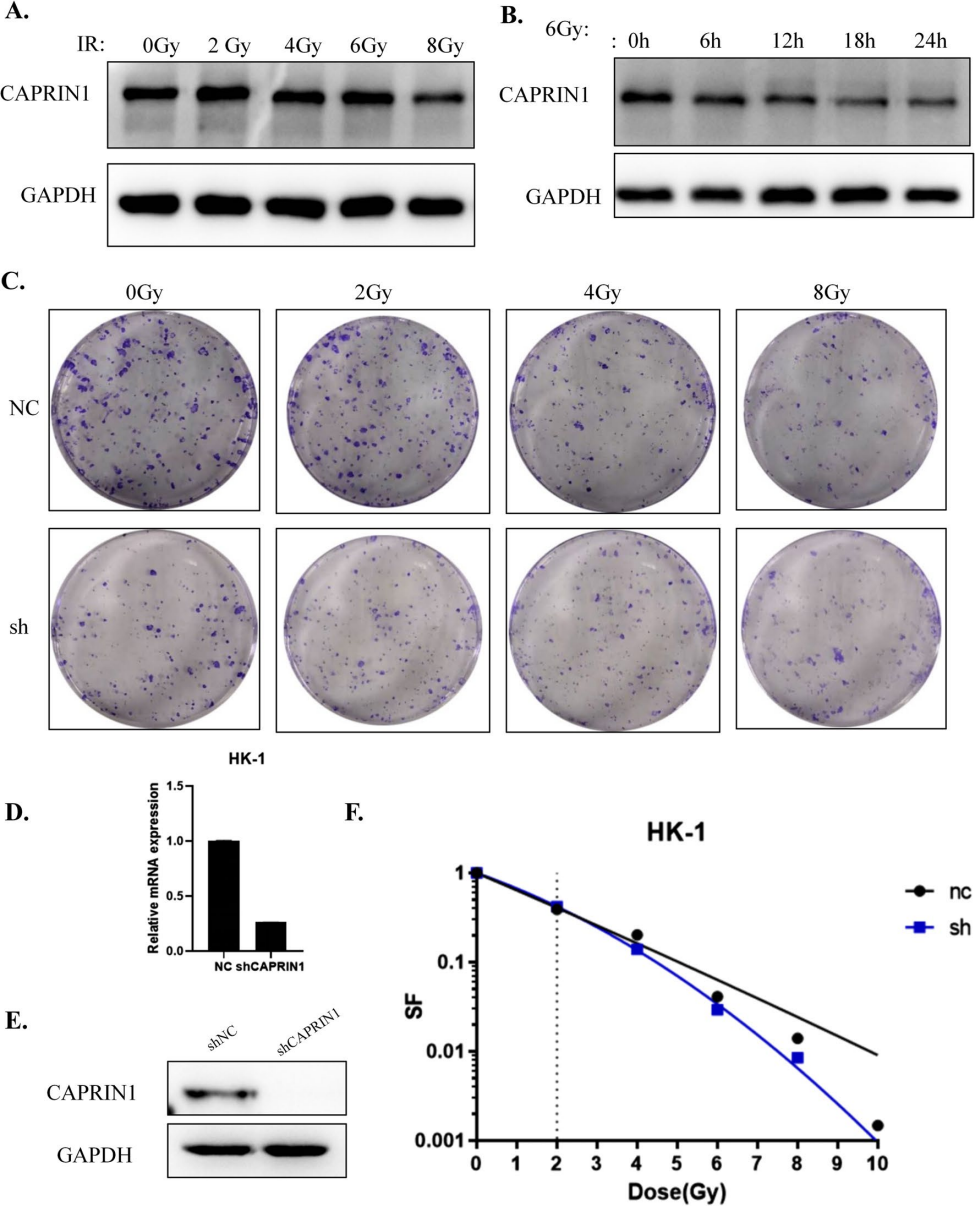
Increased sensitivity of HK-1 cells to X-ray irradiation after CAPRIN1 knockdown. **A** 6-10B cells irradiated with X-rays showed a decrease in CAPRIN1 protein levels with increasing dose. **B** CAPRIN1 protein levels decreased over time at a fixed dose of 6 Gy. **C** Cell survival of HK-1/shCAPRIN1 cells after irradiation at different doses. **D**, **E** mRNA and protein expression in HK-1/shCAPRIN1 cells. **F** Cell survival fraction and survival curve after irradiation of HK-1/shCAPRIN1 cells

Our data have showed that CAPRIN1 knockdown can induce cell apoptosis. We presumed that CAPRIN1 might be correlated with DNA repair and influence the efficacy of radiotherapy, which is currently the treatment of choice for NPC. We therefore irradiated 6-10B NPC cells with X-rays and found that CAPRIN1 levels and the cell survival fraction decreased as the irradiation dose increased (Fig. 5A, C). Meanwhile, CAPRIN1 protein levels decreased over time after treatment at a dose of 6 Gy (Fig. 5B). This finding suggests that CAPRIN1 may be related to the sensitivity of cells to X-rays. To confirm whether other NPC cells have the same reaction, we examined and calculated the cell survival fraction by colony formation assays after X-ray irradiation using the stable CAPRIN1 knockdown NPC cell line HK-1 (Fig. 5D–F). The results showed that CAPRIN1 knockdown in HK-1 cells decreased the cell survival fraction after X-ray irradiation, suggesting that CAPRIN1 may be related to the sensitivity of NPC cells to X-rays.

### Knockdown of CAPRIN1 inhibits the migration of NPC cells

**Fig. 6:**
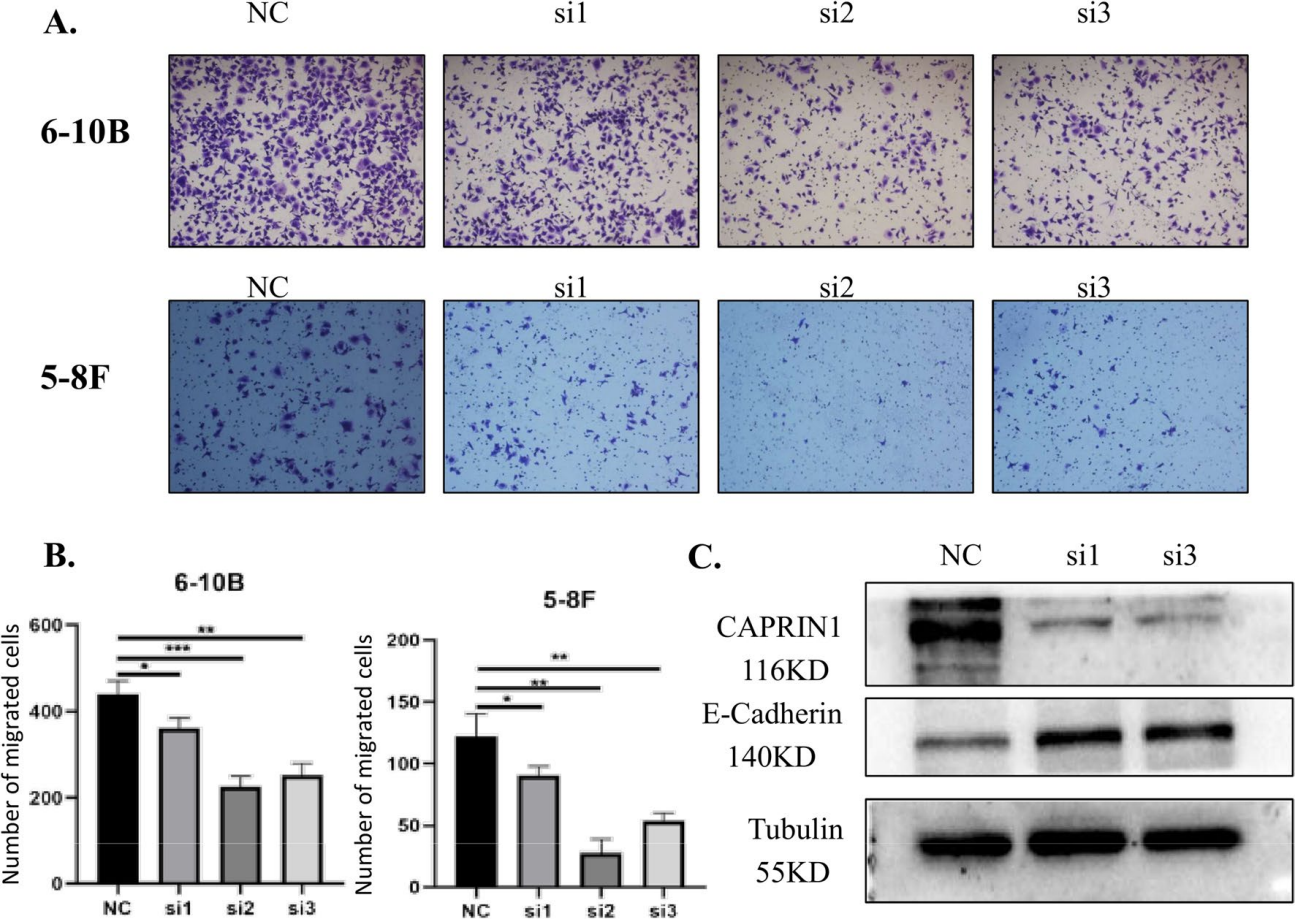
Knockdown of CAPRIN1 in NPC cells inhibits cell migration. **A** Reduced cell migration capacity after CAPRIN1 knockdown in 6-10B and 5-8F cells. **B** Statistical analysis of cell migration after knockdown of CAPRIN1 in 6-10B and 5-8F cells. *p < 0.05, **p < 0.01, ***p < 0.001. **C** The expression of the migration-associated protein E-cadherin in cells with CAPRIN1 knockdown

To investigate whether CAPRIN1 regulates cell migration in NPC, we analyzed cell migration ability by the Transwell assay after CAPRIN1 knockdown (Fig. 6A, B). Here, we used the two same cell lines as the previous experiment, which had good logarithmic growth activity. The results showed that the cell migration ability was reduced after CAPRIN1 knockdown in 6-10B and 5-8F cells. Furthermore, by western blotting, we verified that E-cadherin protein levels decreased after CAPRIN1 knockdown (Fig. 6C), demonstrating that CAPRIN1 is associated with the migration of NPC cells. Thus, CAPRIN1 might be involved in the regulation of NPC cell migration.

## Discussion

In this study, we investigated the roles of SG-related RNA-binding proteins in NPC. We demonstrated that CAPRIN1, an RNA-binding proteins associated with stress granule formation, as a potential target for nasopharyngeal carcinoma. CAPRIN1 was significantly upregulated in nasopharyngeal carcinoma cells and tissues. We characterized the biological functions of CAPRIN1 by knocking down its expression in NPC cells. Our data suggest that CAPRIN1 has a pro-carcinogenic role in NPC, promoting cell proliferation and migration. Importantly, we found that CAPRIN1 knockdown induced cell cycle arrest and apoptosis, conferred drug sensitivity to NPC cells, and influenced the efficacy of radiotherapy. Our data suggest that CAPRIN1 might be a therapeutic target in NPC.

Interestingly, we found that NPC cells showed increased sensitivity to both cisplatin and rapamycin after CAPRIN1 knockdown. In clinical practice, cisplatin is administered to cause DNA damage [20], and rapamycin inhibits the mTOR pathway [21]. Moreover, we verified through colony formation experiments that the sensitivity of tumor cells to X-ray irradiation increased after CAPRIN1 knockdown in NPC cells. Our data suggest that CAPRIN1 may be a potential target for radiotherapy combined with chemical agents.

CAPRIN1 is present in messenger ribonucleoprotein particles (mRNPs), which contain many RNA-binding proteins (RBPs) [22]. Previous studies have shown that CAPRIN1 is part of stress granules, which is functioning like dynamic cytoplasmic reservoirs containing translationally silenced mRNAs that accumulate in response to cellular stress [23]. Previous studies have shown that CAPRIN1 can directly bind to mRNA and regulate cell proliferation in neurons [18]. Moreover, Caprin1-dependent SG formation has been suggested to be associated with docetaxel resistance [24]. Given the close association of CAPRIN1 with stress granules, it is possible that CAPRIN1 influences the response to drug treatment and is related to the regulation of cellular stress. In our experiments, we treated 6-10B NPC cells with DFO, cisplatin and rapamycin in and measured CAPRIN1 protein levels under these stress conditions, the results showed an decrease in CAPRIN1 protein levels in NPC cells. Furthermore, the decrease in stress granules may be related to the drug sensitivity of the cells.

The oncogenic mechanisms of CAPRIN1 might relate to cell cycle regulation in tumor cells. We found that CAPRIN1 knockdown may block NPC cells in S phase. CAPRIN1 has been reported to have mRNA-binding capacity and to bind Cyclin D2 directly [18]. We examined the expression of Cyclin D2 after knocking down CAPRIN1 in 6-10B cells and found that CAPRIN1 knockdown decreased the mRNA and protein levels of Cyclin D2. Our results supported the notion that CAPRIN1 might be involved in cell cycle regulation. Further experimental studies to determine whether CAPRIN1 binds directly to Cyclin D2 mRNA to regulate its mRNA stability are warranted.

In addition to regulating the cell cycle, CAPRIN1 can also regulate apoptosis. It has been reported that the inhibition of stress granule assembly can promote apoptosis [25]. We found an increase in apoptosis after knocking down CAPRIN1 in NPC cells. We observed an upregulation of two key apoptosis-related proteins, cleaved-PARP and cleaved-Caspase 3, suggesting that CAPRIN1 may regulate apoptosis through a Caspase 3-related signaling pathway.

The mechanisms by which CAPRIN1 regulates tumor cell progression and the therapeutic response through stress pathways were not fully elucidated in our study. In the analysis of TCGA data, we found that CAPRIN1 expression is upregulated in a variety of tumors. Recent studies suggest that CAPRIN1 may have an oncogenic role in the development of a variety of types of cancer. It has been reported in prostate cancer that the E3 ubiquitin ligase substrate binding junction SPOP recognizes CAPRIN1 in the cytoplasm and mediates its ubiquitin-mediated degradation; mutated SPOP fails to degrade CAPRIN1, leading to increased abundance and ultimately evoking tumor cell resistance to stress granule inducers such as doxorubicin, sodium arsenite and H_2_O_2_ [24]. In colorectal cancer, it has been reported that miR-193a can cause tumor cell cycle abnormalities and inhibit cell proliferation by targeting CAPRIN1 [26]. Therefore, emerging evidence in other cancers might help elucidate the mechanisms underlying the oncogenic roles of CAPRIN1 in NPC.

Overall, the present study characterized the molecular functions and mechanisms of the SG-related RBP CAPRIN1 in NPC cells. We found that CAPRIN1 promoted cell proliferation and migration and regulated cell cycle progression and apoptosis in NPC cells. Moreover, CAPRIN1 knockdown conferred sensitivity to cisplatin and rapamycin in NPC cells and enhanced the response to X-ray irradiation. CAPRIN1 might be a therapeutic target in NPC.

## Supplementary Information

Below is the link to the electronic supplementary material.

Supplementary file1 (DOCX 24 kb)

Supplementary file2 (TIF 13283 kb)

Supplementary file3 (TIF 12563 kb)

Supplementary file4 (TIF 26545 kb)
