# Supplementary material for: STRESS granule-associated RNA-binding protein CAPRIN1 drives cancer progression and regulates treatment response in nasopharyngeal carcinoma
Source: Med Oncol. 2022 Dec 14;40(1):47. doi: 10.1007/s12032-022-01910-w (PMC9750908; doi:10.1007/s12032-022-01910-w)
Supplement: Supplementary file 1 — Supplementary file1 (DOCX 24 kb) [file 12032_2022_1910_MOESM1_ESM.docx]

**Supplementary Tables and Figure Legends**

**Supplementary Table S1. Primers used in this study**

| gene symbol | 5'-3' Forward Primer | 5'-3' Reverse Primer |
| --- | --- | --- |
| CAPRIN1 | GGAACGAATGAACAAAGGGGA | TGACTTAGTGCCATGAAACTCCT |
| CCND2 | TTTGCCATGTACCCACCGTC | AGGGCATCACAAGTGAGCG |
| GAPDH | TGCACCACCAACTGCTTAGC | GGCATGGACTGTGGTCATGAG |

**Supplementary Table S2. Antibodies used in this study**

| Antibody | Company |
| --- | --- |
| GAPDH | CST, USA |
| TUBULIN | Proteintech, CHINA |
| CAPRIN1 | Proteintech, CHINA |
| CCND2 | Proteintech, CHINA |
| cleaved-PARP | CST CHINA |
| cleaved-Caspase3 | CST CHINA |
| P-S6K | CST CHINA |
| S6K | CST CHINA |

**Supplementary Figures**

Supplementary Figure S1. mRNA expression of CAPRIN1 in NPC cells.

Supplementary Figure S2. CAPRIN1 was up-regulated in multiple tumors in the TCGA dataset.

Supplementary Figure S3. Expression of stress granule formation-associated RNA-binding proteins in nasopharyngeal carcinoma tissues and normal tissues

1. The heatmap to show the mRNA expression of the SG-associated RNA-binding proteins in GSE12452. (B) The heatmap to show the mRNA expression of the SG-associated RNA-binding proteins in GSE53819. (C) Venn diagrams showing the significant up-regulated stress granule formation-associated genes in nasopharyngeal carcinoma GEO datasets. Note, NPC-CONSENSUS denotes up-regulated genes in the GEO2R differential expression analysis of NPC GEO datasets. The stress granule formation-associated genes were the Tier1 list defined in rnagranuledb (<http://rnagranuledb.lunenfeld.ca/>). The curated RBPs were defined by Gerstberger S et al. Nat Rev Genet. 2014 PMID: 25365966.
