# Supplementary figures and images for: STRESS granule-associated RNA-binding protein CAPRIN1 drives cancer progression and regulates treatment response in nasopharyngeal carcinoma
Source: Med Oncol. 2022 Dec 14;40(1):47. doi: 10.1007/s12032-022-01910-w (PMC9750908; doi:10.1007/s12032-022-01910-w)

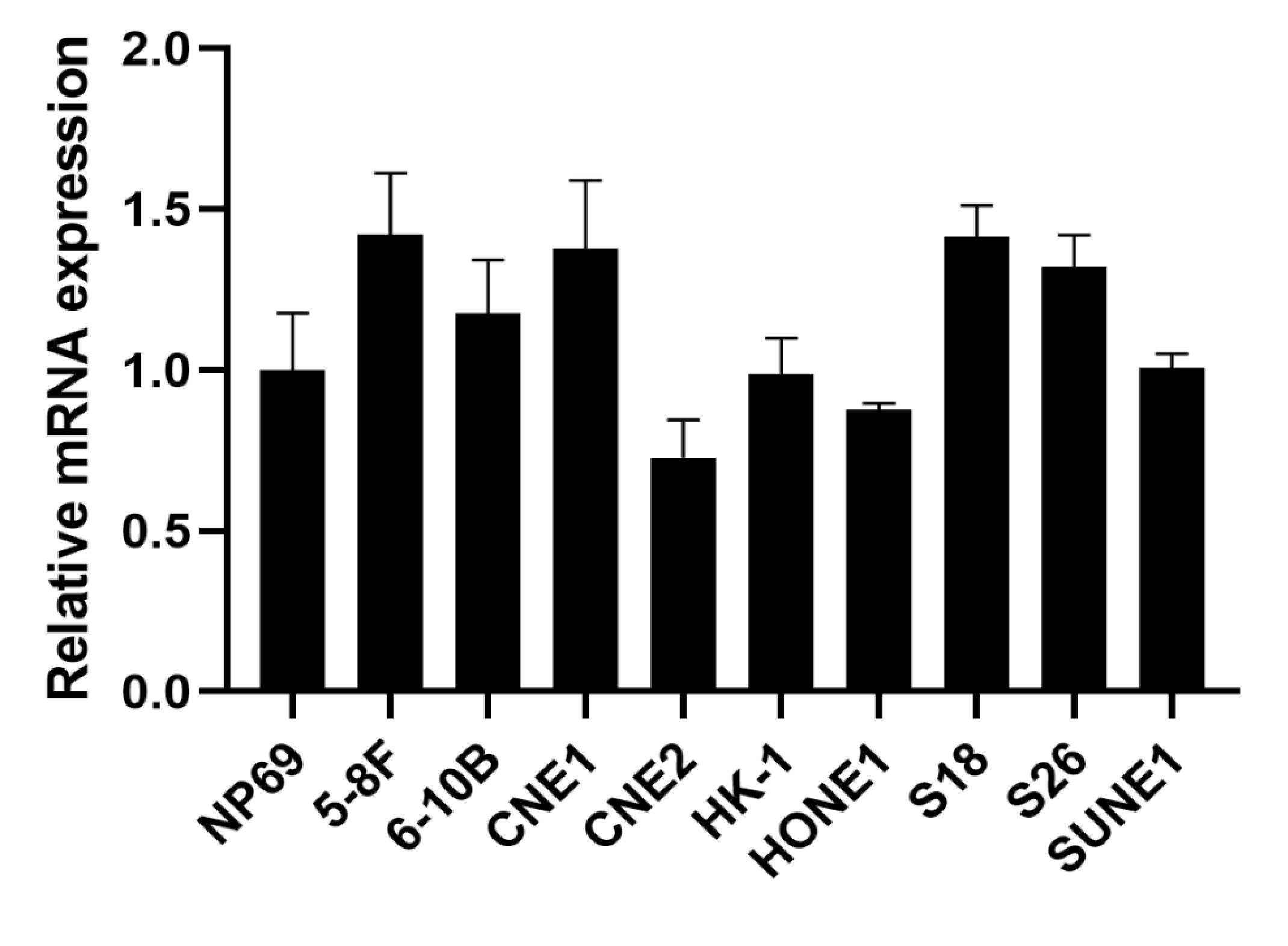

Supplement: Supplementary file 2 — Supplementary file2 (TIF 13283 kb) [file 12032_2022_1910_MOESM2_ESM.tif]

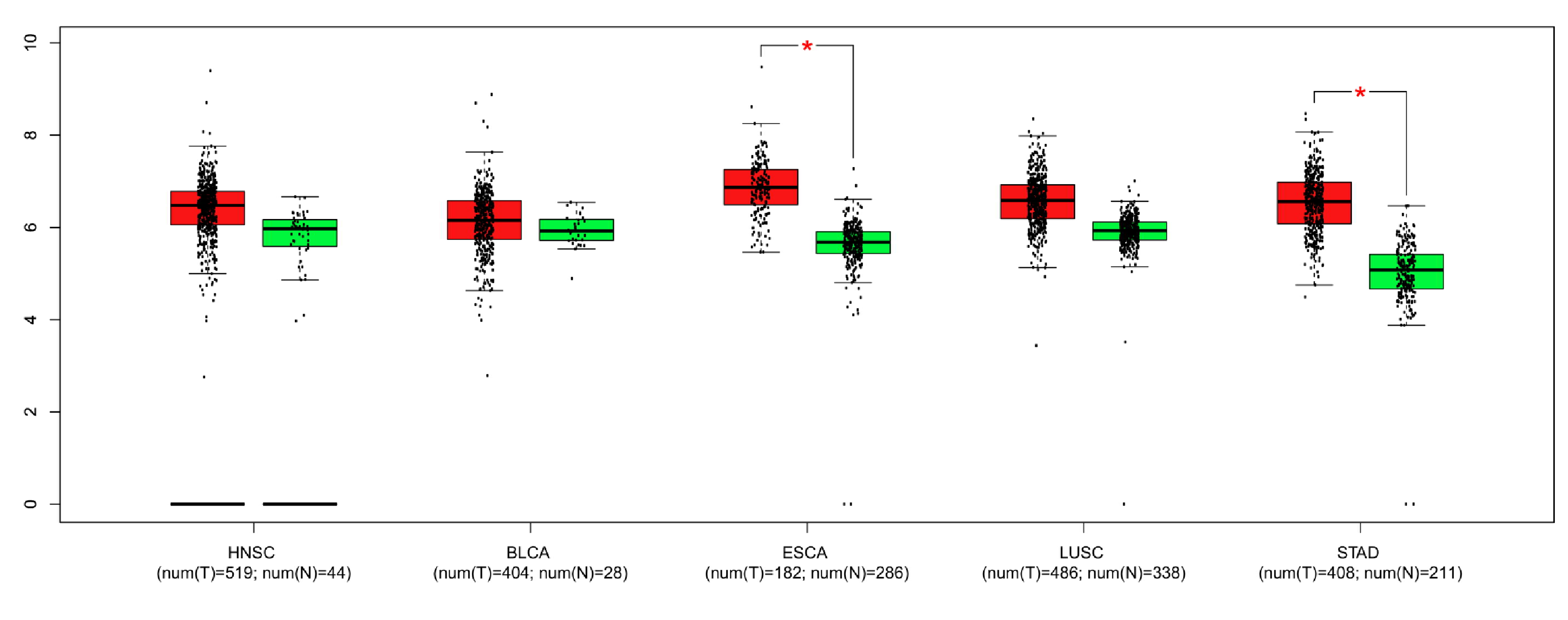

Supplement: Supplementary file 3 — Supplementary file3 (TIF 12563 kb) [file 12032_2022_1910_MOESM3_ESM.tif]

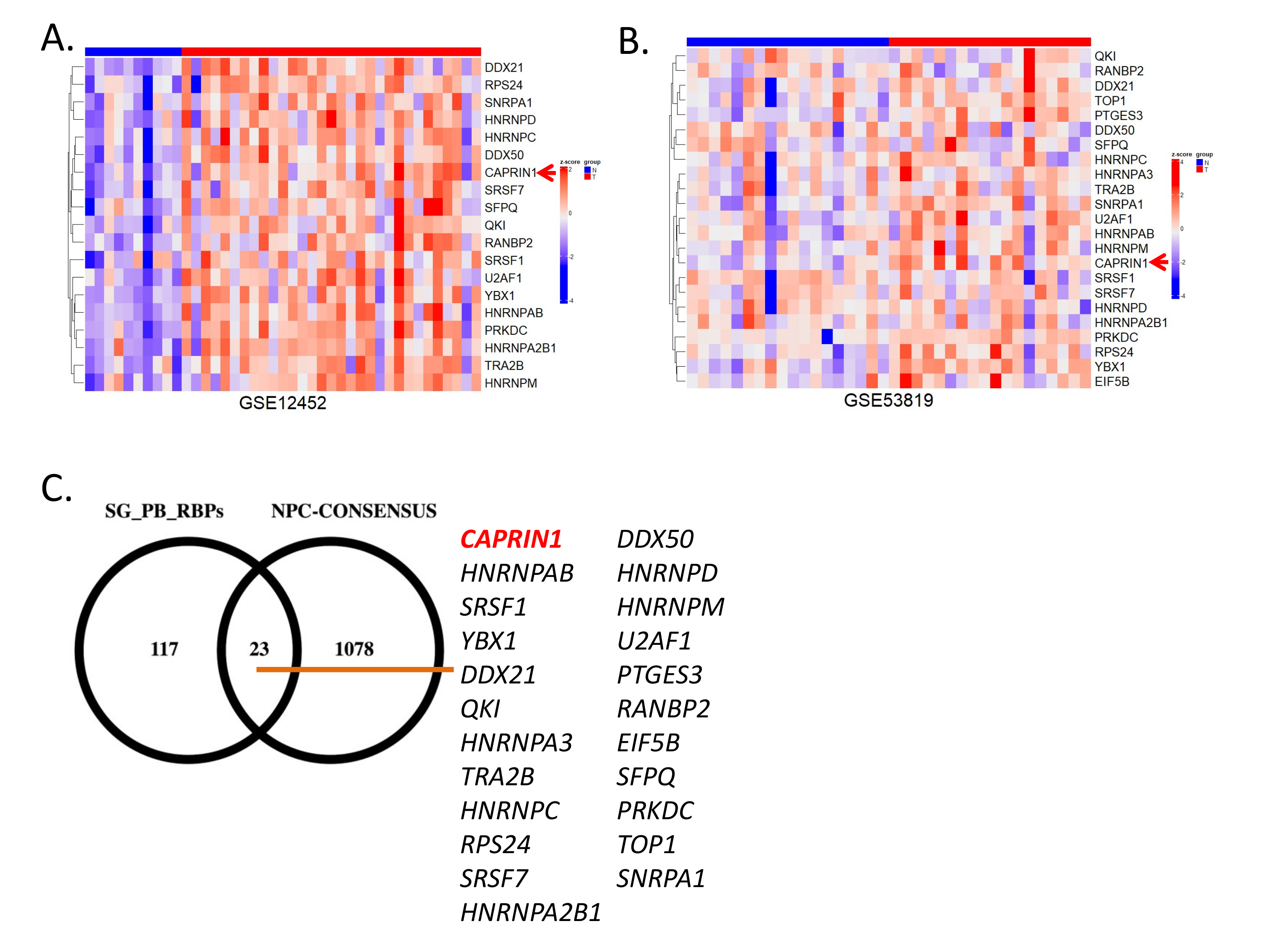

Supplement: Supplementary file 4 — Supplementary file4 (TIF 26545 kb) [file 12032_2022_1910_MOESM4_ESM.tif]
